# Early lexical processing of Chinese one-character words and Mongolian words: A comparative study using event-related potentials

**DOI:** 10.3389/fpsyg.2022.1061990

**Published:** 2023-01-17

**Authors:** Kai Zhang, Feng Gu, Hongzhi Yu

**Affiliations:** ^1^Department of Chinese Language and Literature, Northwest Minzu University, Lanzhou, China; ^2^Key Laboratory of China’s Ethnic Languages and Intelligent Processing of Gansu Province, Northwest Minzu University, Lanzhou, China; ^3^Neurocognitive Laboratory for Linguistics and Semiotics, College of Literature and Journalism, Sichuan University, Chengdu, China; ^4^Key Laboratory of China’s Ethnic Languages and Information Technology of Ministry of Education, Northwest Minzu University, Lanzhou, China

**Keywords:** visual word recognition, Chinese one-character words, Mongolian words, lexical processing, alphabetic language, logographic language

## Abstract

Logographic language and alphabetic language differ significantly in orthography. Investigating the commonality and particularity of visual word recognition between the two distinct writing systems is informative for understating the neural mechanisms underlying visual word recognition. In the present study, we compared the chronometry of early lexical processing and the brain regions involved in early lexical processing between Chinese (logographic language) and Mongolian (alphabetic language) by recording event-related potentials (ERPs) using both implicit and explicit reading tasks. Familiar Chinese one-character words (lexical) and unknown Chinese one-character words (non-lexical) were pseudorandomly presented to native Chinese readers in Experiment 1. Mongolian words (lexical) and pseudowords (non-lexical) were pseudorandomly presented to native Mongolian readers in Experiment 2. In the color decision task, participants were asked to decide the color (black or blue) of each stimulus. In the lexical recognition task, participants were asked to report whether they could recognize each stimulus. The results showed that in both experiments and both tasks, ERPs to lexical items differed significantly from those to non-lexical items in the parietooccipital scalp region approximately 250 ms after stimulus onset, reflecting the early lexical processing, which likely originated from the ventral occipitotemporal cortex as revealed by source analysis. These results indicated that although Chinese and Mongolian differed markedly in orthographic features, the neural mechanisms underlying early lexical processing are similar between the two languages.

## 1. Introduction

There are distinct types of writing systems in the world (Daniels and Bright, 1996) mainly including the alphabetic writing system and the logographic writing system. The former usually uses a few symbols to denote consonants and vowels, i.e., grapheme-phoneme association. The latter usually uses a variety of symbols to denote word meaning, i.e., grapheme-morpheme association. Therefore, whether the neural cognitive processing of alphabetic words and logographic words relies on the same or different mechanisms is an important research topic in the cognitive neuroscience of language. Clarifying this question would be informative for understating the neural mechanisms underlying visual word recognition, especially for evaluating and developing the computational and psychological models of visual word recognition. Previous metabolic-imaging studies found that there was both commonality and particularity regarding the cortical areas involved in reading alphabetic words and logographic words (e.g., Chee et al., 2000; Tan et al., 2001b, 2005a; Thuy et al., 2004; Bolger et al., 2005; Perfetti et al., 2007; Xu et al., 2015). The commonality was reflected by the fact that the ventral occipitotemporal regions in the left hemisphere were involved in word reading in different languages. In particular, the visual word form area (VWFA) in the left fusiform gyrus shows strikingly consistent activation across tasks and languages. The particularity was reflected by the fact that some areas (e.g., the middle frontal gyrus, or MFG) were reported to be activated during the reading of logographic words (Tan et al., 2001a,b, 2005b), and some areas (e.g., the posterior superior temporal gyrus, or pSTG) were reported to be activated during reading alphabetic language (Paulesu et al., 2000). For instance, Tan et al. (2001b) observed that the left inferior frontal lobe and the left temporal-occipitoparietal junction were involved in reading both alphabetic and logographic words, whereas the left lateral middle frontal cortex and some regions in the right hemisphere (e.g., the right frontal operculum, the right superior and inferior parietal lobules, and the right visual system) were specifically activated during the reading of logographic words (Chinese one-character words). The involvement of the brain areas in the right hemisphere was explained by the fact that reading Chinese characters requires extensive analysis of the spatial information because Chinese one-character words are visually complex symbols.

In addition to the brain regions involved in word recognition, many studies have investigated the chronometry of lexical processing, which is usually investigated by comparing the brain responses [e.g., event-related potentials (ERPs), event-related fields (ERFs), and intracranial recordings] elicited by words and those elicited by orthographically-matched pseudowords (e.g., Sereno and Rayner, 2000; Chen et al., 2013; Shtyrov and MacGregor, 2016) or comparing the brain responses elicited by high-frequency words and those elicited by low-frequency words (e.g., Assadollahi and Pulvermuller, 2003; Hauk and Pulvermuller, 2004; Proverbio et al., 2008). The results usually showed that the early brain responses (100–250 ms) were modulated by lexicality (words vs. pseudowords) or word frequency (high-frequency words vs. low frequency words), reflecting the rapid lexical processing of written words. However, the chronometry of lexical processing varied from 100 ms to 250 ms across different studies. This is because lexical processing is affected by several factors: (1) length of stimulus [i.e., how many letters in a word (e.g., Hauk and Pulvermuller, 2004; Xue et al., 2008)]; (2) upper case or lower case of the letters (e.g., Ellis et al., 2007; Vergara-Martínez et al., 2020); (3) duration of stimulus presentation (e.g., Ellis et al., 2007; Xue et al., 2008); (4) experimental task (implicit or explicit reading tasks) (e.g., Sereno et al., 1998; Kiefer and Martens, 2010; Chen et al., 2013); (5) age of participants (e.g., Maurer et al., 2006; Tong et al., 2016); and (6) printed or hand-written words (e.g., Hellige and Adamson, 2007; Vergara-Martinez et al., 2021). In the present study, we aimed to match the experimental settings and stimulus parameters between two experiments to investigate the commonality and particularity between the early lexical processing of logographic words and alphabetic words.

Although the chronometry of lexical processing is affected by the factors listed above, a difference in ERP/F between words and pseudowords (or between high-frequency words and low-frequency words) of approximately 250 ms in the parietooccipital scalp region has been consistently observed in many studies (e.g., Hauk et al., 2006; Dufau et al., 2015; Strijkers et al., 2015; Vergara-Martinez et al., 2021; Yu et al., 2022). This early ERP difference reflecting lexical processing was also observed in a recent study using Chinese one-character words as stimuli, which are logographic words (Yu et al., 2022), implying that the ERP difference at approximately 250 ms in the parietooccipital scalp region might be a common signature of lexical processing for both alphabetic and logographic words. In the present study, we tested this hypothesis by comparing the lexical processing of Chinese one-character words (logographic words) in Experiment 1 and Mongolian words (alphabetic words) in Experiment 2 using the same experimental procedures.

Chinese is special because it is almost the only logographic language in the current world (Cheng, 1989; Daniels and Bright, 1996; Tan et al., 2001b). The Chinese language uses thousands of characters to describe meaning rather than pronunciation. Almost every character is a lexical item that has pronunciation and meaning (Yeh and Li, 2002; Xue et al., 2019). Written Chinese is among the extremely deep orthographies because the grapheme-phoneme mapping is arbitrary (Xu et al., 2015). In contrast, Mongolian is an alphabetic language. Like Italian and Finnish (Bolger et al., 2005), Mongolian is among the shallow orthographies because the grapheme-phoneme mapping is transparent. Moreover, Mongolian words are string-like and are written from top to bottom (Daniels and Bright, 1996). To this end, Chinese one-character words and Mongolian words are ideal for investigating the commonality and particularity of lexical processing between alphabetic language and logographic language.

**Figure 1:**
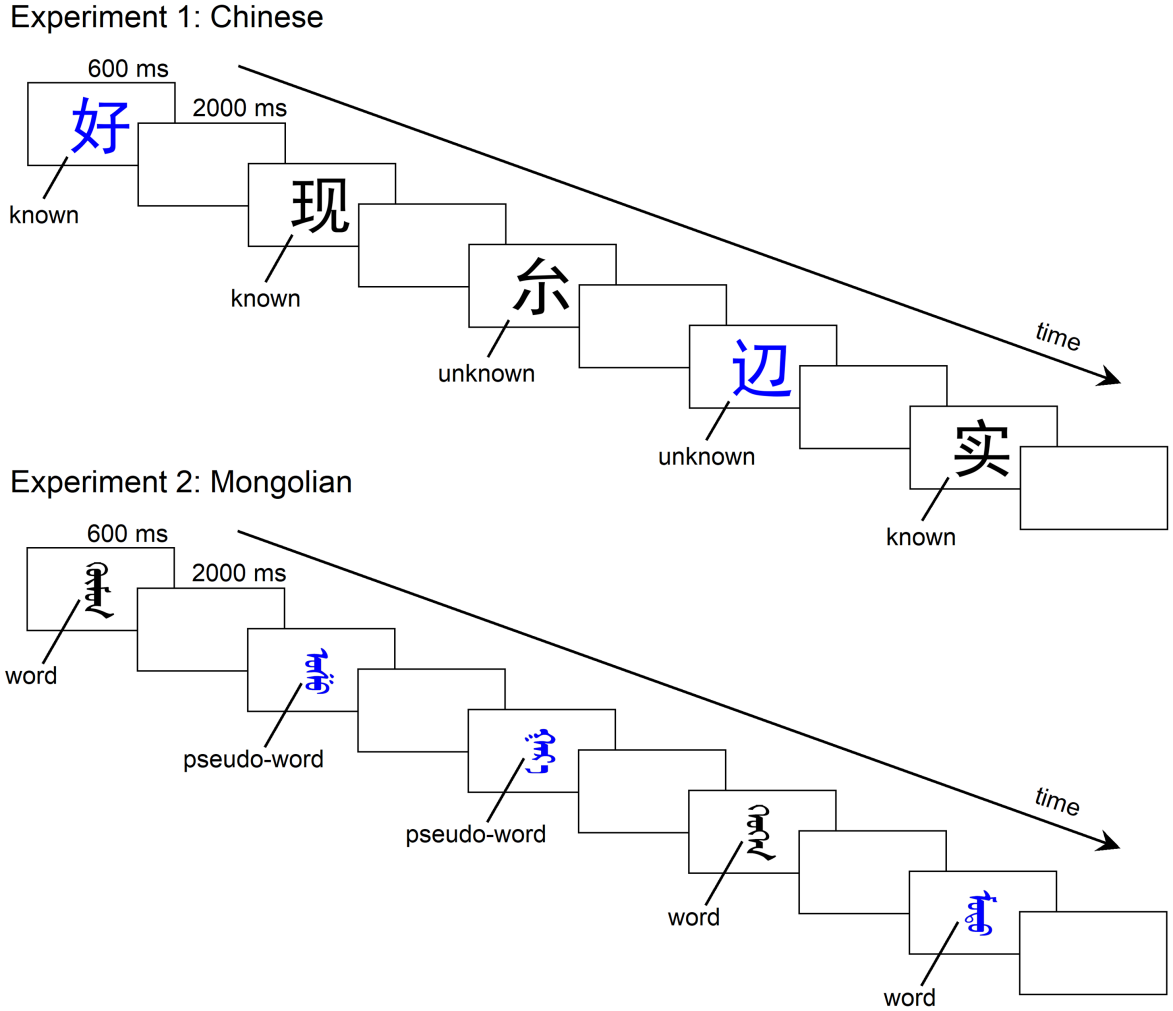
Illustration of the experimental procedures. There were two tasks in both experiments: a color decision task and a lexical recognition task. The stimuli were pseudorandomly presented in the center of a monitor with an inter-stimulus interval (offset to onset) of 2,000 ms. The duration of presentation for each stimulus was 600 ms. In the color decision task, participants were asked to determine the color of each stimulus as quickly as possible by pressing two buttons (one for blue and the other for black). In the lexical recognition task, participants were asked to press two buttons to indicate whether s/he knew the stimulus.

In the present study, we adopted the widely used implicit reading task (color decision) and explicit reading task (lexical decision/recognition) in previous research. By using both implicit and explicit tasks, we were able to investigate both automatic and controlled lexical processing. In Experiment 1 (Chinese), 15 known one-character words (lexical items) and 15 unknown one-character words (non-lexical items) were used as stimuli. The known one-character words were commonly used characters, whereas the unknown one-character words were of very low frequency that the participants did not know them. All the stimuli were repeatedly and pseudorandomly presented to participants (Figure 1). In the color decision task, participants were asked to decide the color (black or blue) of each stimulus. In the lexical recognition task, participants were asked to report whether they could recognize each stimulus. In Experiment 2 (Mongolian), 15 words (lexical items) and 15 pseudowords (non-lexical items) were used as stimuli. The experimental procedures were the same as those in Experiment 1. In both experiments, the ERPs to lexical items and those to non-lexical items were recorded and compared. The chronometry of lexical processing would be reflected by the ERP difference between lexical items and non-lexical items. We expected an ERP difference of approximately 250 ms in the parietooccipital scalp region for both experiments, reflecting early lexical processing of both alphabetic and logographic words.

## 2. Methods

### 2.1. Participants

Twenty native Chinese readers (11 males and 9 females) participated in Experiment 1. The participants learnt English as second language at school age and did not learn any other languages. The participants were aged from 21 to 29 years (Mean = 24.0, SD = 2.3) and were undergraduate or graduate students studying at Northwest Minzu University. Another 20 native Mongolian readers (11 males and 9 females) participated in Experiment 2. The participants learnt Chinese and English as second languages at school age and did not learn any other languages. The participants were aged from 21 to 30 years (*M* = 25.1, SD = 2.8) and were also undergraduate or graduate students studying at Northwest Minzu University. There were no significant differences in “age” [*t*(38) = 1.554, *p* = 0.137, two-tailed] or “years of education” [*t*(38) = 0.383, *p* = 0.706, two-tailed] between the two groups of participants. All participants were right-handed as assessed by the Edinburgh Inventory (Oldfield, 1971). All participants had normal or corrected to normal vision. All participants reported no color blindness and no history of mental illness. All participants were asked to read and sign the informed consent form before the experiment and were paid after finishing the experiment. The experimental procedures were approved by the Ethics Committee of Northwest Minzu University.

### 2.2. Stimuli

In Experiment 1, 15 common Chinese one-character words were used as lexical items (Table 1). The character frequency ranged from 19.9 to 8881.9 per million (mean = 1033.4 per million), which were assessed according to the SUBTLEX-CH frequency list (Cai and Brysbaert, 2010). The other 15 very low-frequency characters were used as non-lexical items. The character frequency ranged from 0 to 0.2 per million (mean = 0.02 per million). All participants reported after the experiment that they did not know these very low-frequency one-character words. Thus, the high-frequency one-character words were called known one-character words and the very low-frequency one-character words were called unknown one-character words. The stimuli were grouped into 15 pairs to match the visual and orthographic characteristics between the two groups of stimuli. Each pair had a known one-character word and an unknown one-character word, and the two characters were closely matched in visual or orthographic characteristics because the two characters differed only in one component (e.g., “王” in “现” and “山” in “岘”). The different components in the known one-character words and the unknown one-character words were matched in component frequencies (an index of visual familiarity) and stroke numbers (an index of visual complexity).

**Table 1:** Stimuli.

Experiment 1						Experiment 2					
Known one-character words			Unknown one-character words			Words			Pseudowords		
	Pronunciation	Meaning		Pronunciation	Meaning		Pronunciation	Meaning		Pronunciation	Meaning
现	[xian2]	Now	岘	NA	NA	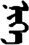	niɡe	One	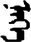	nege	NA
映	[ying2]	Mirror	旼	NA	NA	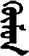	xümün	People	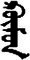	kümün	NA
好	[hao3]	Good	矷	NA	NA	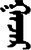	ɣɑǰɑr	Place	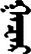	ɣɑzɑr	NA
张	[zhang1]	Open	枨	NA	NA	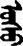	usu	Water	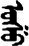	ušu	NA
故	[gu4]	Event	敀	NA	NA	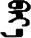	bey-e	Body	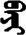	beyi	NA
实	[shi2]	Fact	宩	NA	NA	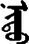	nidü	Eye	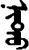	nitü	NA
朵	[duo3]	A quantifier	尕	NA	NA	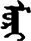	mori	Horse	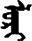	lori	NA
允	[yun3]	Allow	厼	NA	NA	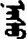	nasu	Age	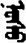	nosu	NA
否	[fou3]	No	夻	NA	NA	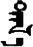	tɑl-ɑ	Grassland	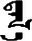	dɑl-ɑ	NA
志	[zhi4]	Ambition	忎	NA	NA	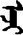	eǰi	Mother	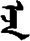	eǰe	NA
闪	[shan3]	Flash	闬	NA	NA	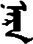	nɑrɑ	Sun	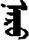	nɑru	NA
式	[shi4]	Style	弎	NA	NA	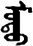	modu	Tree	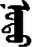	nodu	NA
团	[tuan2]	Group	囝	NA	NA	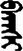	bɑɣšɪ	Teacher	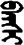	banšɪ	NA
疗	[liao2]	Cure	疔	NA	NA	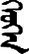	xeüxen	Girl	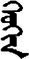	xeüxin	NA
边	[bian1]	Edge	辺	NA	NA	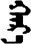	χɑr-ɑ	Black	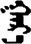	ɣɑr-ɑ	NA

Fifteen known one-character words (lexical items) and fifteen unknown one-character words (non-lexical items) were used as stimuli in Experiment 1. Fifteen Mongolian words (lexical items) and fifteen pseudowords (non-lexical items) were used as stimuli in Experiment 2. For both experiments, the stimuli were grouped in pairs to match the visual or orthographic characteristics between the two groups of stimuli (see main text). The pronunciations of Chinese one-character words were given in Chinese pinyin. The pronunciations of Mongolian words and pseudowords were given in Latin transcription (School of Mongolian Studies Inner Mongolia University, 1999). The lexical meanings of known Chinese one-character words and Mongolian words are given. NA, not available.

The stimuli of Experiment 2 consisted of 15 Mongolian words (lexical items) and 15 Mongolian pseudowords (non-lexical items) (Table 1). According to the DaBartel and BaoJirimutu (1999), the words were frequently used in Mongolian (ranging from 45.4 to 8402.1 per million, mean = 1112.6 per million). The stimuli were grouped into fifteen pairs to match the visual or orthographic characteristics between the two groups of stimuli. Each pair had a word and a pseudoword, and the two stimuli were closely matched in visual or orthographic characteristics because the two words differed only in one letter (vowel or consonant). The pseudowords were all orthographically legal in Mongolian. The methods of creating the pseudowords were based on previous research (e.g., Proverbio and Adorni, 2008).

### 2.3. Procedure

Experiment 1 consisted of two tasks: a color decision task and a lexical recognition task. For the color decision task, the 30 stimuli listed in Table 1 were pseudorandomly presented in the center of a monitor with an inter-stimulus interval (offset to onset) of 2,000 ms. The duration of presentation for each stimulus was 600 ms. Stimuli were presented in *Heiti* font against a white background. In each block, each one-character word was repeated 10 times (half in blue and the other half in black). The 300 stimuli in total were pseudorandomly presented with the following restrictions: (1) Any one-character word did not appear consecutively; (2) the same color was repeated at most three times; and (3) the same lexical status (lexical or non-lexical) was repeated at most three times. The participants were asked to determine the color of each stimulus as quickly as possible by pressing two buttons (one for blue and the other for black) using the index and the middle fingers of their right hand. During the experiment, participants sat in front of the monitor in a dimly lit and sound-attenuated chamber. The view distance was 150 cm. The size of the stimulus was approximately 8 × 8 cm (width × height, 3° × 3° in visual angle). For the lexical recognition task, the stimuli were presented the same as in the color decision task (Figure 1), but the participants were instructed to press two buttons to indicate whether s/he knew the stimulus instead of determining the color of the stimulus. The two buttons registering the known one-character word and unknown one-character word were counterbalanced across the participants. Each task consisted of two blocks. Thus, each participant engaged in four blocks. The order of the four blocks (CLLC or LCCL, C for the color decision task and L for the lexical recognition task) was counterbalanced across the participants. There were short between-block breaks for rest. The whole experiment including the application and removal of the electric cap lasted approximately 1.5 h. E-Prime 3.0 was used for stimulus presentation and behavioral data recording.

The experimental procedure of Experiment 2 was the same as Experiment 1 except that the stimuli were replaced by the 30 Mongolian words and pseudowords (Table 1). The stimuli were presented in *IeaUnicode*, a common font of Mongolian scripts. The visual size of each stimulus ranged from approximately 5 × 7 cm (1.9° × 2.7° in visual angle) to 5 × 10 cm (1.9° × 3.8° in visual angle).

In the present study, each stimulus (lexical or non-lexical) was repeated 40 times during the whole experiment to collect enough trials to obtain the ERPs. Massive stimulus repetition may affect the brain response elicited by the stimulus. However, Yu et al.’s (2022) study demonstrated that 40 repetitions of each stimulus did not affect the ERP signature of early lexical processing in comparison with four repetitions.

### 2.4. Electroencephalogram recording

The electroencephalogram (EEG) signal was recorded with a SynAmps 2 amplifier (NeuroScan, Charlotte, NC, United States) from 64 Ag/AgCl electrodes secured in an elastic cap. According to the extended international 10/20 system, electrodes were placed in corresponding positions on the scalp. The electrical activities of the left and right mastoids were recorded. The vertical EOG was recorded by two electrodes placed above and below the left eye. The electrode AFz between FPZ and FZ served as the grounding electrode. All recording electrodes were referenced to the tip of the nose. The electrode impedance was kept below 5 kΩ. Continuous EEG data (0.03–100 Hz) were recorded with a sampling rate of 500 Hz.

### 2.5. Electroencephalogram data analysis

The EEG data of the two experiments were analyzed in the same way. The specific steps are as follows: (1) The EEG data were digitally bandpass (0.1–25 Hz) filtered using a finite impulse response filter; (2) the blink artifacts were corrected by adopting a regression-based procedure (Semlitsch et al., 1986); (3) the continuous EEG data were segmented into 600-ms epochs, including a 100-ms pre-stimulus baseline; (4) baseline correction was applied using the 100-ms baseline; (5) epochs with extreme amplitudes exceeding ±50 μV in any channel (excluding the vertical EOG channel) were rejected, and the remaining EEG epochs were averaged to obtain the ERP; (6) the ERPs to lexical items and those to non-lexical items were obtained for each task; and (7) The ERPs were rereferenced using the common average (the average of all 64 electrodes).

### 2.6. N170 analysis

The N170 peak latencies and mean amplitudes were analyzed. The N170 peak latencies were determined as the time points when the ERP amplitude reached peak activity at approximately 170 ms at electrodes PO7 and PO8. To calculate the mean N170 amplitudes, an electrode cluster in the left parietooccipital scalp region (P7, P5, PO7, PO5, O1) and an electrode cluster in the right parietooccipital scalp region (P8, P6, PO8, PO6, O2) were selected. The mean ERP amplitudes of the five electrodes in each cluster were calculated within a 40-ms time-interval (138–178 ms). The time-interval was determined by means of the “Collapsed Localizers,” which center at the peak of the N170 in the averaged ERPs across conditions (Luck and Gaspelin, 2017).

### 2.7. Mass univariate analysis of event-related potentials

For each experiment, the ERPs of lexical items and the ERPs of non-lexical items were compared by repeated measures, two-tailed *t*-tests at all 64 electrodes (except the vertical EOG channel) and at each sampling point from 50 to 500 ms (i.e., 14,400 total comparisons). The Benjamini–Hochberg procedure (Benjamini and Hochberg, 1995) was used to control the false discovery rate (FDR), which refers to the mean proportion of significant test results that are actually false discoveries. The FDR level was set to 5%. The Mass Univariate ERP Toolbox was used for the *t*-tests with FDR controls (Groppe et al., 2011).

### 2.8. Lexical effect around 200–250 ms

According to the *a priori* knowledge, the early lexical processing is likely reflected by the ERP amplitude around 200–250 ms in the parietooccipital scalp region. To further investigate whether the early lexical processing was modulated by the factors of “task,” “hemisphere,” and “experiment,” the mean ERP amplitude of the difference ERP (lexical items minus non-lexical items) were obtained for each participant and each task. The mean ERP amplitude within the time window of 200–250 ms in the left electrode cluster (P7, P5, PO7, PO5, O1) and that in the right electrode cluster (P8, P6, PO8, PO6, O2) were calculated, respectively.

### 2.9. Source analysis

The grand-averaged difference ERPs for each task and each experiment was analyzed by low-resolution electromagnetic tomography (LORETA) (Pascual-Marqui et al., 1994) using the four-shell ellipsoidal head model in BESA Research (ver. 7.1, BESA GmbH, Germany^1^).

## 3. Results

### 3.1. Behavioral results

Table 2 shows the mean hit rates and the mean response times for each condition and each experiment. The mean response times were calculated from the trials with correct responses. In the color decision task, the hit rate refers to the percentage of presentations in which the participants correctly identified the color of the stimulus. In the lexical recognition task, the hit rate refers to the percentage of presentations in which the participants’ responses were in accordance with the lexical status (known and unknown) of the stimulus.

**Table 2:** Behavioral results.

	Experiment 1 (Chinese)			Experiment 2 (Mongolian)		
		Mean hit rate (%)	Mean response time (ms)		Mean hit rate (%)	Mean response time (ms)
Color decision	Known one-character words	98.0 (range: 95.0–100.0)	508.3 (SD = 87.4)	Words	98.0 (range: 95.0–100.0)	613.0 (SD = 181.3)
	Unknown one-character words	98.0 (range: 95.0–100.0)	511.6 (SD = 87.5)	Pseudowords	97.0 (range: 92.0–100.0)	628.3 (SD = 190.4)
Lexical recognition	Known one-character words	96.0 (range: 89.0–100.0)	562.3 (SD = 135.9)	Words	95.0 (range: 85.0–100.0)	692.3 (SD = 166.1)
	Unknown one-character words	83.0 (range: 79.0–100.0)	625.9 (SD = 124.5)	Pseudowords	94.0 (range: 81.0–100.0)	718.5 (SD = 163.5)

For the color decision task, a paired-samples *t*-test revealed that Mongolian participants (Experiment 2) spent significantly more time determining the color of the pseudowords than the words [*t*(19) = 2.796, *p* = 0.012, two-tailed]. The same paired-samples *t*-test test performed in Experiment 1 on Chinese participants did not show a significant difference [*t*(19) = 1.171, *p* = 0.256, two-tailed].

For the lexical recognition task, paired-samples *t*-tests revealed that both Chinese participants (Experiment 1) and Mongolian participants (Experiment 2) spent significantly more time determining non-lexical items than lexical items [Chinese: *t*(19) = 8.595 *p* < 0.001, two-tailed; Mongolian: *t*(19) = 3.690, *p* = 0.002, two-tailed].

### 3.2. N170 results

**Figure 2:**
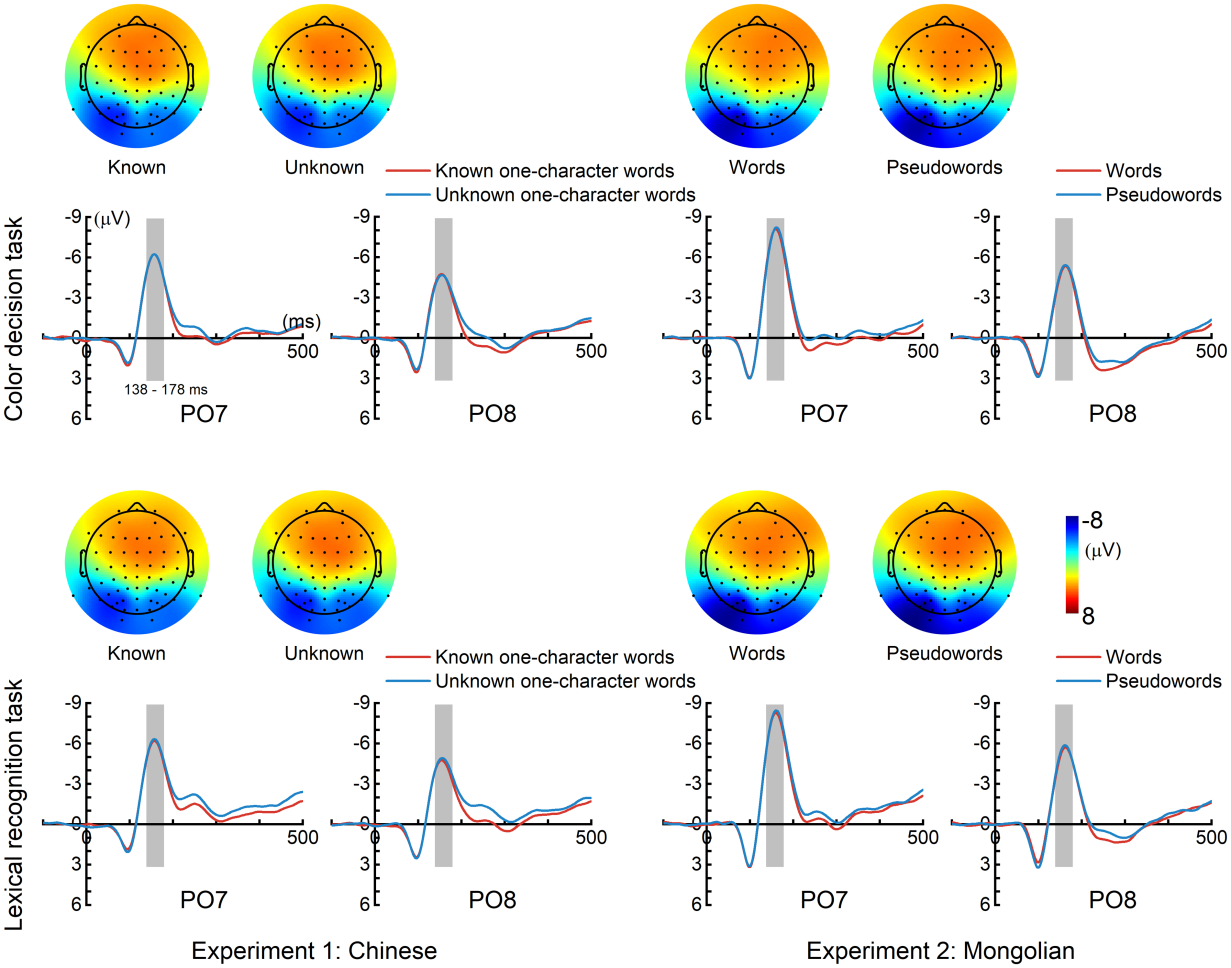
Grand-averaged event-related potentials (ERPs). A prominent N170 response was elicited in each condition and each experiment. The time interval (138–178 ms), represented by gray bars was used for calculating the mean N170 amplitudes. The topographic maps (mean magnitudes within 138–178 ms) of N170 for each condition are displayed.

Figure 2 displays the grand-averaged ERPs for each condition and each experiment. A left hemisphere lateralized N170 response was elicited in each condition and each experiment, which showed the largest amplitude around PO7 and reversed polarity in the frontal scalp region.

**Figure 3:**
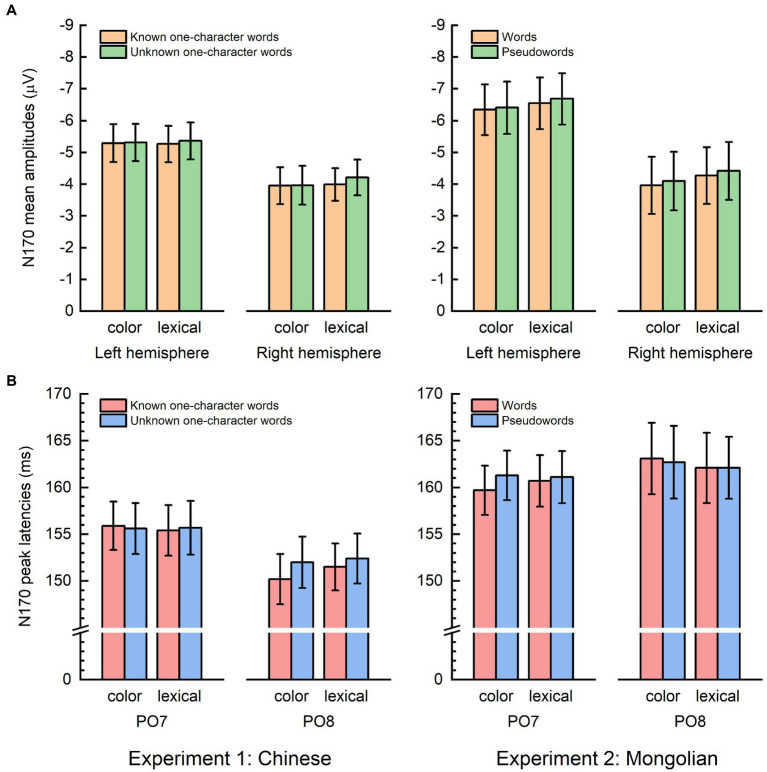
Mean N170 amplitudes and N170 peak latencies. **(A)** The mean N170 amplitudes elicited by lexical items were significantly smaller than those elicited by non-lexical items. The mean N170 amplitudes were significantly larger in the left hemisphere compared with the right hemisphere. **(B)** The N170 peak latencies were significantly shorter for lexical items than in non-lexical items in the right hemisphere (PO8) for Chinese but not Mongolian. The error bar represents one standard error of the mean.

Figure 3 shows the mean N170 amplitudes and the N170 peak latency results. The mean N170 amplitudes were analyzed using “lexicality” (lexical items and non-lexical), “task” (color decision and lexical recognition), and “hemisphere” (left and right) as within-subjects factors, and “experiment” (Experiment 1 and Experiment 2) as a between-subjects factor. There was a significant main effect of lexicality [*F*(1, 38) = 5.821, *p* = 0.021] indicating that the mean N170 amplitudes for the lexical items were significantly smaller than those for the non-lexical items. There was also a significant main effect of hemisphere [*F*(1, 38) = 10.897, *p* = 0.002], indicating that the mean N170 amplitudes were significantly larger in the left hemisphere than in the right hemisphere. No other main effects or significant interactions were observed (*P*s > 0.100).

The N170 peak latencies were also analyzed using “lexicality” (lexical and non-lexical), “task” (color decision and lexical recognition), and “hemisphere” (left and right) as within-subjects factors, and “experiment” (Experiment 1 and Experiment 2) as a between-subjects factor. There was a significant interaction across lexicality, hemisphere, and experiment [*F*(1, 38) = 6.150, *p* = 0.018]. *Post-hoc* analysis indicated that the interaction was driven by the fact that the N170 peak latencies were significantly shorter for the lexical items than for the non-lexical items in the right hemisphere (PO8) for Experiment 1 [*F*(1, 19) = 7.648, *p* = 0.012] but not for Experiment 2 [*F*(1, 19) = 0.248, *p* = 0.624]. There was no significant main effect for task [*F*(1, 19) = 0.012, *p* = 0.914], and no other significant interactions (*P*s > 0.125).

### 3.3. Mass univariate analysis results

**Figure 4:**
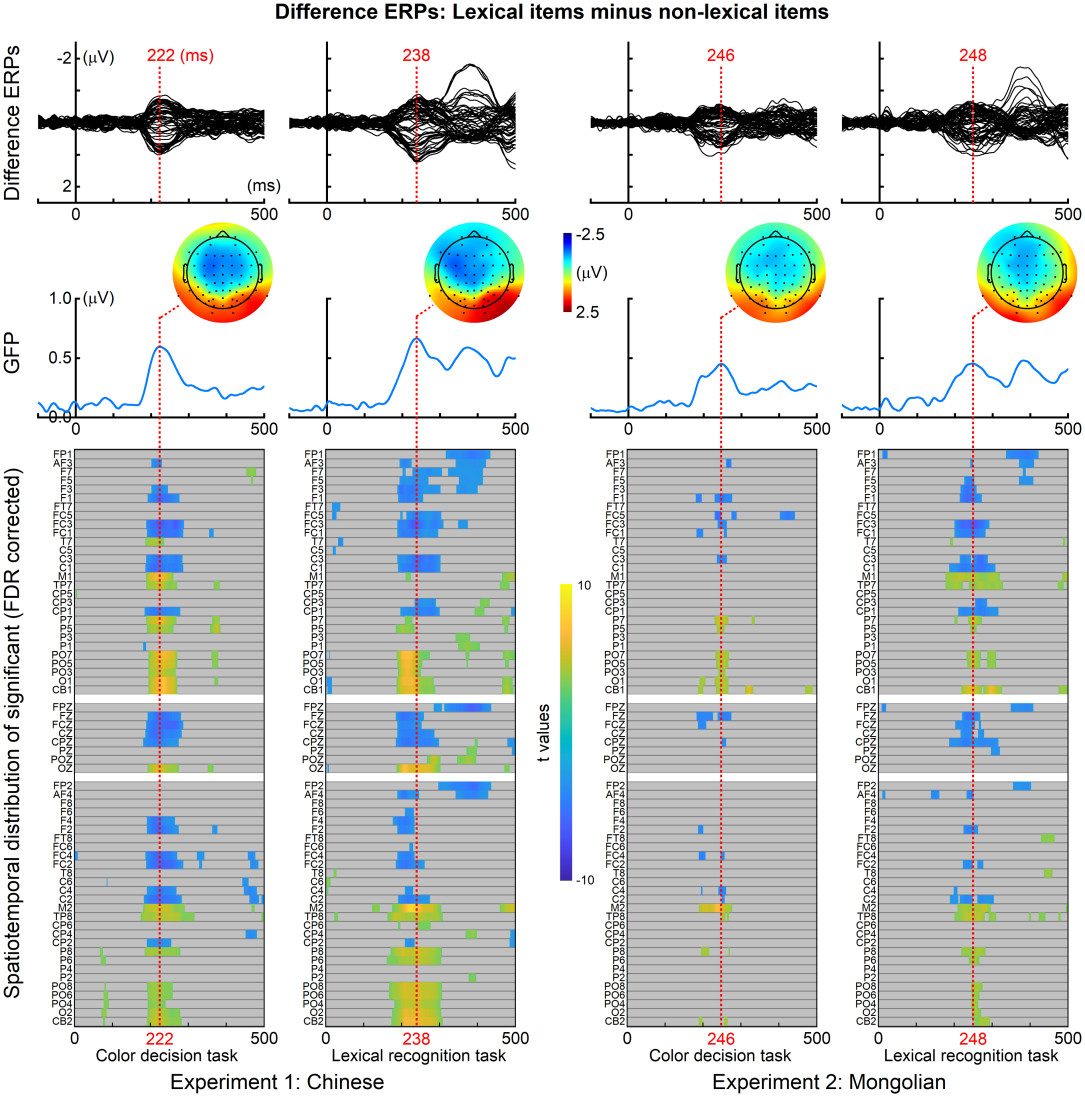
Event-related potentials differences between lexical and non-lexical items. For both tasks and both experiments, significant differences between the ERPs of lexical items and the ERPs of non-lexical items were observed at approximately 200–250 ms (highlighted by red dotted lines). The difference ERPs was obtained by subtracting the ERPs of non-lexical items from the ERPs of lexical items. The global field power (GFP) of the difference ERPs, and the topographic maps at the GFP peaks are illustrated.

For each task in each experiment, the ERPs of lexical items were compared with the ERPs of non-lexical items. The resultant spatiotemporal distributions of significance with FDR correction for multiple comparisons are illustrated in Figure 4. The difference ERPs (grand-averaged) obtained by subtracting the ERPs of non-lexical items from the ERPs of lexical items, the global field power (GFP) of the difference ERPs, and the topographic maps at the GFP peaks are shown in Figure 4. The difference ERPs was illustrated by superimposing the ERPs from all 64 channels (i.e., butterfly plot). Significant differences between the ERPs of lexical items and the ERPs of non-lexical items were consistently observed at approximately 200–250 ms for both tasks in both experiments (highlighted by red dotted lines in Figure 4).

### 3.4. Lexical effect around 200–250 ms

**Figure 5:**
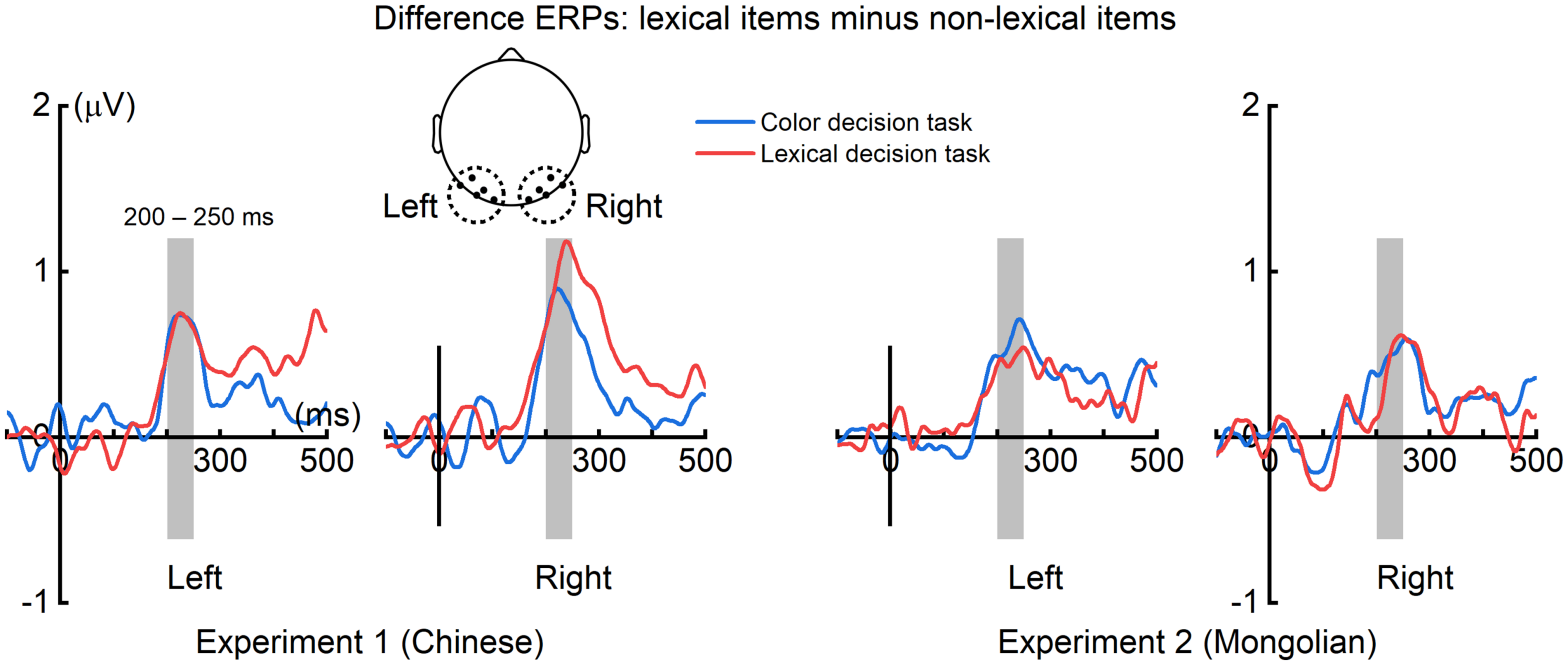
Difference ERPs in the left and right scalp regions. For both tasks and both experiments, prominent difference ERPs (obtained by subtracting the ERPs of lexical items from the ERPs of non-lexical items) were observed at approximately 200–250 ms in the left and right scalp regions.

**Figure 6:**
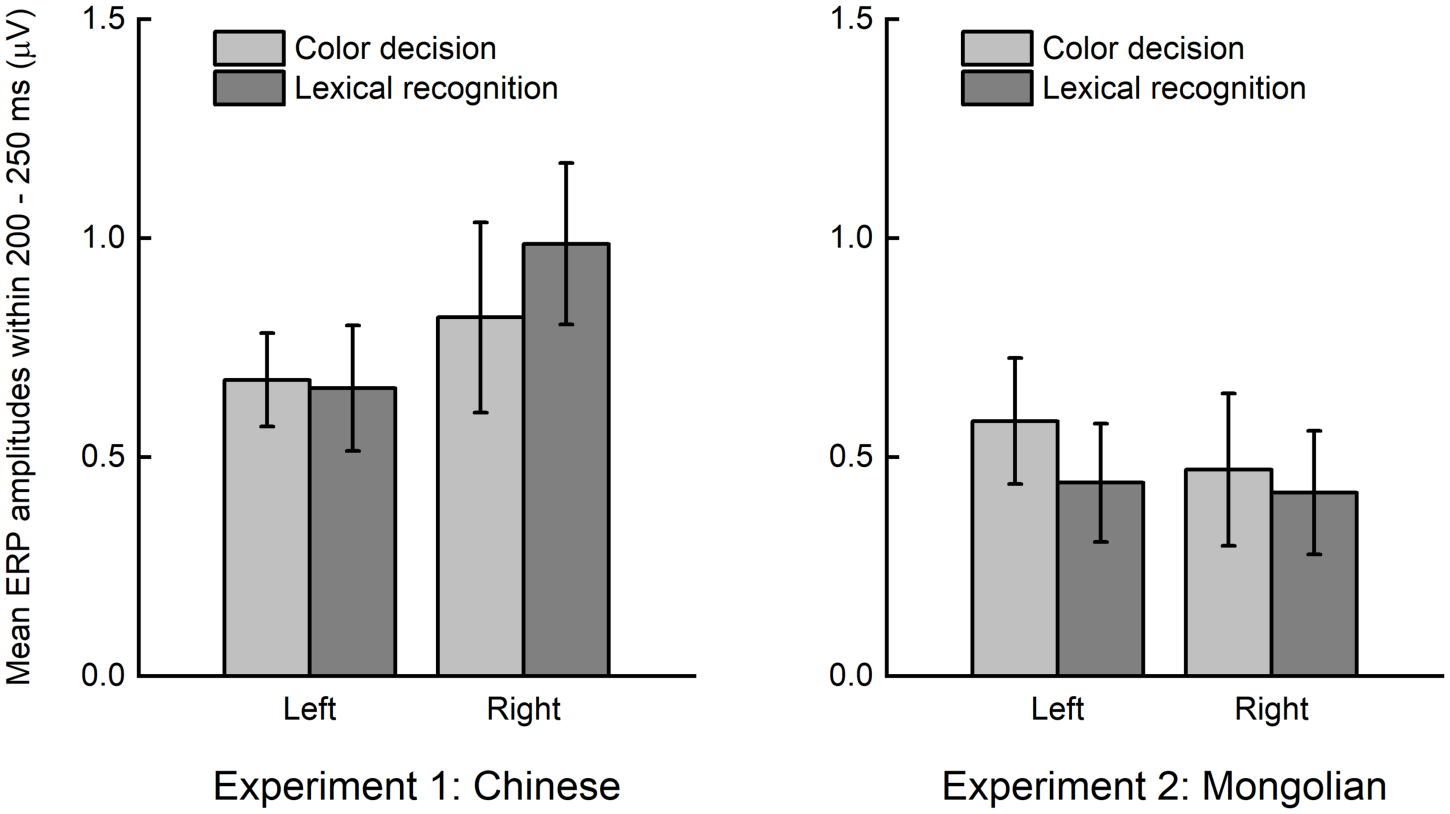
Lexical effect around 200–250 ms. The early lexical effects were not significantly modulated by the factors of “task” (color decision and lexical recognition) and “hemisphere” (left and right), but significantly different between Experiment 1 (Chinese) and Experiment 2 (Mongolian).

The grand averaged difference ERPs (obtained by subtracting ERPs of non-lexical items from those of lexical items) in the left (averaged across P7, P5, PO7, PO5, O1) and right (averaged across P8, P6, PO8, PO6, O2) scalp regions for each experiment and each task were illustrated in Figure 5. The mean ERP amplitude within 200–250 ms were analyzed by ANOVA using “task” (color decision and lexical recognition) and “hemisphere” (left and right) as within-subjects factors, and “experiment” (Experiment 1 and Experiment 2) as a between-subjects factor (Figure 6). There was a significant main effect of “experiment” [*F*(1, 38) = 4.151, *p* = 0.049], indicating the early lexical effect was more prominent for Chinese compared with Mongolian. There was no any other significant main effects nor significant interactions between/across factors (*P*s > 0.241).

### 3.5. Source analysis results

**Figure 7:**
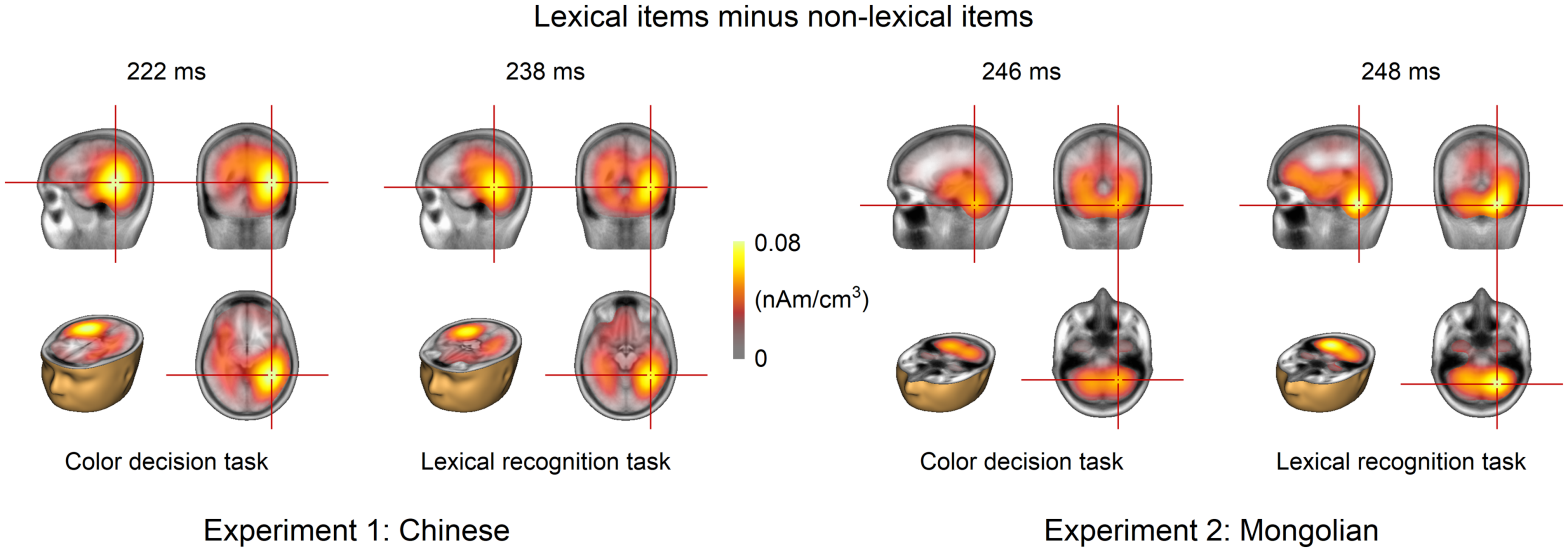
Source analysis results. Source analysis was performed at the GFP peak of the grand-averaged ERPs for each task and each experiment. The left and right ventral occipitotemporal cortices were the potential neural origins for both tasks and both experiments.

The grand-averaged difference ERP for each task in each experiment was analyzed using LORETA. The results at the GFP peaks are shown in Figure 7. Cerebral activities around the left and right ventral occipitotemporal cortices were consistently observed for both tasks and both experiments. The peak activities were consistently located around the right ventral occipitotemporal cortex.

## 4. Discussion

The early ERP signatures of lexical processing are modulated by several factors such as word length, duration of stimulus presentation, and the level of task demand. Therefore, it can hardly compare results across different studies with different stimulus characteristics and experimental settings. In the present study, we compared the chronometry of lexical processing between Chinese and Mongolian individuals using the same experimental procedures. Chinese is a representative language of the logographic writing system, whereas Mongolian is a representative language of the alphabetic writing system. Despite the distinct orthography difference between the two languages, we found that the prominent ERP signature (i.e., the ERP difference between lexical items and non-lexical items at approximately 200–250 ms in the parietooccipital scalp regions) reflecting lexical processing was consistent between the two languages, suggesting similar neural mechanisms of lexical processing between alphabetic and logographic words.

### 4.1. Lexical processing at approximately 200–250 ms

The results of Experiment 1 (Chinese) in the present study were highly consistent with a recent study that found ERP amplitude differences between known one-character words and unknown one-character words of approximately 220 ms in the parietooccipital scalp region and N170 peak latency differences between known one-character words and unknown one-character words at PO8 (Yu et al., 2022). The experimental procedures were the same between the current study and Yu et al.’s (2022) study, except that different stimuli were used. Experiment 2 (Mongolian) in the present study also found a significant ERP difference between lexical items and non-lexical items at approximately 200–250 ms in the parietooccipital scalp region, which was generally consistent with the results of Experiment 1 (Chinese). Thus, the results of the present study revealed the commonality of the neural mechanisms underlying visual word recognition between alphabetic and logographic languages (i.e., early automatic lexical processing approximately 200–250 ms after stimulus onset in the ventral occipitotemporal cortex). Notably, Mongolian words are written vertically. The present results found that the orientation of the written scripts likely did not affect the early ERP effect of lexical processing, which was consistent with previous study using Hebrew words that are written from the right to the left (Nemrodov et al., 2011).

The topographic maps of the difference ERP (lexical minus non-lexical) at approximately 200–250 ms (i.e., parietooccipital distribution, see Figure 4) and the source analysis results (ventral occipitotemporal area) suggested that the ERP difference at approximately 200–250 ms likely reflected orthographic processing of the lexical items, because the ventral occipitotemporal area is a well-established region to be involved in orthographic processing (McCandliss et al., 2003; Dehaene and Cohen, 2011; Woolnough et al., 2021). The word forms of the lexical items were represented as long-term memories in the brain, i.e., the orthographic lexicon (Coltheart et al., 2001; Taylor et al., 2013), but the word forms of the non-lexical items were not. Thus, the ERP difference at approximately 200–250 ms likely reflected the activation of long-term memory for familiar word forms. This view is consistent with previous studies which suggested that access to orthographic word forms was reflected by the P200 in the parietooccipital scalp region or the N250 in the frontocentral scalp region (e.g., Barnea and Breznitz, 1998; Liu and Perfetti, 2003; Carreiras et al., 2005; Grainger et al., 2006; Holcomb and Grainger, 2007; Wu et al., 2012; Bermudez-Margaretto et al., 2020). Because the latency of P200 and N250 varies greatly across different studies, we suggested that the difference ERP obtained by subtracting ERPs of pseudowords from ERPs of words would be a better way to reveal the ERP signature of lexical orthographic processing. The results of the present study suggested that the orthographic word forms for different writing systems (alphabetic and logographic) are likely represented in the same brain area (the ventral occipitotemporal area) and accessed at the same time course (200–250 ms) during visual word recognition. Moreover, the significant ERP difference at approximately 200–250 ms was observed in both implicit and explicit reading tasks (Figure 6), suggesting that lexical orthographic processing was automatic and irrespective of the readers’ attention to the stimuli. Furthermore, the results of both Experiment 1 (Chinese) and Experiment 2 (Mongolian) suggested that, although not statistically significant (Figure 6), lexical orthographic processing tended to be right hemisphere lateralized (Figures 4–7). However, according to Yu et al. (2022), the right hemisphere tendence might be a result of stimulus repetition because each stimulus was repeated 40 times.

### 4.2. Lexical processing in the N170 time window

The N170 amplitude was consistently observed to show the coarse print tuning effect. That is, the N170 elicited by word-like stimuli is enhanced in amplitude and left hemisphere lateralized compared with non-orthographic stimuli such as false-font strings (Bentin et al., 1999; Maurer et al., 2006; Cao and Zhang, 2011; Tong et al., 2016). Nevertheless, N170 amplitude seldom reflects lexical effects. Only a few studies observed N170 amplitude differences between words and pseudowords or between high-frequency words and low-frequency words (Sereno et al., 1998). The present study found a significant lexical effect on N170 (i.e., the N170 elicited by lexical items were significantly smaller than that elicited by non-lexical items) (Figure 3A). However, this effect was weak, and reached statistical significance only when data from both experiments were submitted to ANOVA. The weak effect might be the reason why the lexical effect on N170 amplitude was seldom observed in previous studies. N170 is supposed to be modulated by predictive coding (Price and Devlin, 2011; Zhao et al., 2019; Huang et al., 2022). The pseudowords might be associated with a larger prediction error and thus resulted in enhanced N170 amplitudes compared with words.

The present study also found a significant N170 peak latency difference between known one-character words and unknown one-character words at PO8 in Experiment 1 (Chinese). This result was consistent with Yu et al.’s (2022) study. However, the lexical effect reflected by the N170 peak latency difference was not reported by previous studies and was not found in Experiment 2 (Mongolian) of the present study. Therefore, the N170 peak latency difference between lexical and non-lexical items may be specific to Chinese lexical processing. Another potential reason is that the N170 peak latency difference would be observed only when simple or short stimuli (e.g., monosyllabic words) were used, reflecting a facilitated neural response to lexical items.

### 4.3. Phonological and semantic processing of written words

The phonological word forms for spoken words are represented in the left perisylvian areas (Pulvermuller, 2001; Pulvermuller and Fadiga, 2010), which are automatically accessed approximately 150–200 ms after the word recognition point even during passive listening (e.g., Pulvermuller et al., 2001; Shtyrov et al., 2010, 2011; for a reviews see Pulvermuller et al., 2009; Shtyrov et al., 2011). The orthographic representations of written words (i.e., orthographic lexicon) are supposed to be associated with their phonological representations (i.e., phonological lexicon) in many cognitive models of visual word recognition (Coltheart et al., 2001; Taylor et al., 2013), and some studies using the priming paradigm suggested that the phonological information of written words is implicitly accessed during visual word recognition (Ferrand and Grainger, 1992; Rastle and Brysbaert, 2006). However, the present ERP study and previous ERP/F studies did not observe any activation of the perisylvian area where the phonological word forms are represented (e.g., Chen et al., 1995). Additionally, spoken words usually did not activate their orthographic representations in the ventral occipitotemporal cortex (e.g., Shtyrov et al., 2010; MacGregor et al., 2012). We speculated that the orthography-phonology association might hardly be detected by using ERP/F recordings.

Previous studies using spoken words as stimuli suggested that the neural representations of word meanings are distributed over the cerebral cortex (for reviews see Pulvermuller, 2001; Pulvermuller et al., 2009; Pulvermuller and Fadiga, 2010). The semantic representations of words are automatically and rapidly (<200 ms) activated by spoken words (e.g., Pulvermuller et al., 2004; Pulvermüller et al., 2005). The present study did not observe any ERP signature that reflected semantic access, because the semantic representations of the lexical items that represented in different areas in the cerebral cortex could not be revealed after averaging the brain responses to all the lexical items. Moreover, the implicit and explicit reading tasks used in the present study forced participants to focus on the color or the lexicality of the stimuli rather than word meaning. This might be an additional reason that semantic processing was not observed in the present study.

## 5. Conclusion

The present study found that early lexical processing of both Chinese one-character words and Mongolian words was reflected by the ERP difference between lexical items and non-lexical items at approximately 200–250 ms in the parietooccipital scalp region. Source analysis results suggested that the ERP difference likely originated from the ventral occipitotemporal area, a region involved in orthographic processing. Therefore, the results of the present study suggested that the lexical orthographic processing of both alphabetic and logographic visual words takes place approximately 200–250 ms after word onset in the ventral occipitotemporal area.

## Data availability statement

The raw data supporting the conclusions of this article will be made available by the authors, without undue reservation.

## Ethics statement

The studies involving human participants were reviewed and approved by Ethics Committee of Northwest Minzu University. The patients/participants provided their written informed consent to participate in this study.

## Author contributions

KZ and HY designed the study. KZ collected and analyzed the data. KZ and FG wrote the original manuscript. HY reviewed and edited the manuscript. All authors contributed to the article and approved the submitted version.

## Conflict of interest

The authors declare that the research was conducted in the absence of any commercial or financial relationships that could be construed as a potential conflict of interest.

## Publisher’s note

All claims expressed in this article are solely those of the authors and do not necessarily represent those of their affiliated organizations, or those of the publisher, the editors and the reviewers. Any product that may be evaluated in this article, or claim that may be made by its manufacturer, is not guaranteed or endorsed by the publisher.

## Footnotes

^1^ http://www.besa.de/
